# ASSOCIATIONS OF SOCIAL RELATIONSHIPS WITH INFLAMMATORY, CARDIOVASCULAR AND IMMUNE CELL BIOMARKERS

**DOI:** 10.1093/geroni/igae098.3807

**Published:** 2024-12-31

**Authors:** Ahmed Ragab, Jiachen Chen, Yumeng Cao, Margaret Doyle, Joanne Murabito, Kathryn Lunetta

**Affiliations:** Boston University, Boston, Massachusetts, United States; Boston University School of Public Health, Boston, Massachusetts, United States; Boston University, Boston, Massachusetts, United States; University of Vermont Larner College of Medicine, Colchester, Vermont, United States; Chobanian and Avedisian School of Medicine, Boston University, Boston, Massachusetts, United States; Boston University, Boston, Massachusetts, United States

## Abstract

Social relationships are essential for health, as social isolation and loneliness are linked to higher risks of morbidity and mortality. These associations may result from behavioral changes, physiological alterations, increased inflammation, and a weakened immune system. Community-based cohorts investigating the association between social isolation and inflammation have not included immune cell phenotypes and have been limited to a small number of inflammatory and cardiovascular biomarkers. We investigated the relationship between the Social Network Index (SNI), and 3 biomarker datasets: 1) 68 inflammatory biomarkers (N=875), 2) 71 cardiovascular biomarkers (N=3295), and 3) 28 immune cell phenotypes(N=996), among Framingham Heart Study Offspring participants (mean age 61). SNI was calculated as the sum of four components: marital status, the presence of close friends or relatives, attendance at religious services, and participation in social groups and treated as an ordinal predictor (1=lowest level of connections, 4= highest). We used Linear Mixed Effects models with SNI as the predictor and biomarkers/immune phenotypes as the outcomes, adjusting for age and sex and CMV (immune phenotypes). We used FDR≤0.05 to declare significance within each biomarker dataset. SNI was positively associated with inflammatory biomarkers HGF, IL-6, OSM, and IL-10RB. SNI was also positively associated with cardiovascular proteins CNTN1, IGFBP3, FGF23, CD56, sRAGE, and IGF1, and negatively associated with GDF15, MMP9, FBN, MCP1, CD14, A1M, IGFBP2, CRP, EFEMP1, and PPBP. SNI was not significantly associated with any of the immune cell phenotypes in the full sample. We conclude that social isolation is associated with multiple inflammatory and cardiovascular biomarkers.

